# Influence of Physical Activity and Nutrition on Obesity-Related Immune Function

**DOI:** 10.1155/2013/752071

**Published:** 2013-11-07

**Authors:** Chun-Jung Huang, Michael C. Zourdos, Edward Jo, Michael J. Ormsbee

**Affiliations:** ^1^Department of Exercise Science and Health Promotion, Florida Atlantic University, 777 Glades Road, FH11A-126B, Boca Raton, FL 33431, USA; ^2^Department of Kinesiology and Health Promotion, California State Polytechnic University, Pomona, Pomona, CA, USA; ^3^Department of Nutrition, Food and Exercise Sciences, The Florida State University, Tallahassee, FL, USA

## Abstract

Research examining immune function during obesity suggests that excessive adiposity is linked to impaired immune responses leading to pathology. The deleterious effects of obesity on immunity have been associated with the systemic proinflammatory profile generated by the secretory molecules derived from adipose cells. These include inflammatory peptides, such as TNF-**α**, CRP, and IL-6. Consequently, obesity is now characterized as a state of chronic low-grade systemic inflammation, a condition considerably linked to the development of comorbidity. Given the critical role of adipose tissue in the inflammatory process, especially in obese individuals, it becomes an important clinical objective to identify lifestyle factors that may affect the obesity-immune system relationship. For instance, stress, physical activity, and nutrition have each shown to be a significant lifestyle factor influencing the inflammatory profile associated with the state of obesity. Therefore, the purpose of this review is to comprehensively evaluate the impact of lifestyle factors, in particular psychological stress, physical activity, and nutrition, on obesity-related immune function with specific focus on inflammation.

## 1. Introduction

The global epidemic of obesity is irrefutably a major public health issue largely because of its comorbidities, namely, cardiovascular disease, type II diabetes, and cancer. Nevertheless, the prevalence of obesity has drastically escalated by nearly 57% over the previous two decades [1, 2]. The National Health and Nutrition Examination Survey (NHANES 2009-2010) reported that 36% of US adults are currently classified as obese (BMI ≥ 30 kg/m^2^), while 16% represent incidences of severe cases (BMI ≥ 35 kg/m^2^) [2]. Based on projections derived from previous NHANES data, 86% of U.S adults will be overweight (BMI ≥ 25 kg/m^2^) or obese (obesity accounting for 51.1%) by 2030 if this epidemiological trend remains unresolved [3–5]. 

Over the past two decades, adipose tissue has been established as a multifunctional organ playing a critical role not only in lipid/energy storage but also in endocrine and immune functions [6–8]. The confirmed presence of secretory molecules derived from adipocytes, such as proinflammatory cytokines (e.g., tumor necrosis factor-alpha [TNF-*α*] and interleukin-6 [IL-6]), constitutes the unique endocrine function of adipose and provides valuable pathophysiological insight regarding obesity and its comorbidities [7]. During unhealthy weight gain, the influx and storage of excess lipids in adipocytes perturbs normal cell function, which consequently induces the overexpression and hyper-secretion of inflammatory peptides into circulation [9]. As a result, obesity is now recognized as a state of low-grade systemic inflammation characterized by high circulating levels of inflammatory molecules, such as TNF-*α*, IL-6, and C-reactive protein (CRP) [10, 11]. Due to the deleterious effects of systemic inflammation on multiple organ tissues, unhealthy weight gain is associated with high risk of developing serious health conditions, such as type II diabetes and cardiovascular disease [7, 12]. Given the involvement of adipose tissue in the inflammatory process, especially in excessive states, it becomes an important clinical objective to identify lifestyle factors that may affect the obesity-immune system dynamic. For instance, stress, physical activity, and nutrition have each shown to be significant lifestyle factors influencing the inflammatory profile associated with the state of obesity [13–15]. Of particular interest, it is well documented that chronic inflammation is also highly correlated to nutritional factors such as the type and amount of carbohydrates, proteins and fats that are consumed in the diet [16–18]. Therefore, the purpose of this review is to comprehensively evaluate the impact of lifestyle factors, in particular psychological stress, physical activity, and nutrition, on obesity-related immune function with special focus on inflammation. 

## 2. Stress and Obesity 

Stress in the body is established through some type of stressor(s), either physical or psychological. When stimulated, the human body responds in a complex manner, incorporating the intertwined activity of the endocrine and nervous systems (hypothalamic-pituitary-adrenal [HPA] and sympathoadrenal [SA] axes). Stress hormones such as cortisol from HPA axis and catecholamines (epinephrine (EPI) and norepinephrine (NE)) from the SA axis have been shown to alter immune cell responses, and this important immune system response coordinates a number of the body's adaptations to the stressor. Notably, elevations in stress hormones (cortisol and catecholamines) are thought to have detrimental effects on the immune system, leading to an imbalance between innate (immediate antigen-nonspecific defense) and adaptive immunity (specific response to a particular foreign antigen creating immunological memory) via the release of immune mediators such as cytokines [19]. In response to acute stress, the innate immune promptly prepares to provide immune protection followed by adaptive immunity when exposed to repeated or prolonged stress, whereas chronic stress can suppress these immune defenses. This stress-immune interaction is an important antiviral defense and fosters the elimination of invading microorganisms [20, 21].

Research has shown that stress induces changes in immune cell distribution [22–25], which ensures that the body's immune response is efficient or elicits an effective immunoprotection. An appropriate distribution of peripheral immune cells provides for the performance of surveillance and effector functions of the immune system [26]. The release of catecholamines and cortisol in response to stressors (physical or psychological) can mediate changes in the immune cell distribution [27, 28]. In response to acute stress, immune response is primarily regulated by catecholamines [24]. This is further supported by other studies demonstrating transient immune cell redistribution via beta-adrenergic activation following acute mental stress [23, 27, 29, 30]. Specifically in response to exercise, monocytes and NK cells (innate immunity) exhibit the greatest fluctuation followed by CD3+ T cells and CD19+ B cells (adaptive immunity) [31–33]. These alterations in immune cells have been shown to correspond with the release of catecholamines [34]. In addition, our laboratory recently found that following a combined physical and psychological stress, NE area-under-the-curve (AUC) was negatively correlated with the percentage of CD19+ B cells, and heart rate (HR) was negatively associated with the percentage change in the CD4/CD8 ratio [35]. These elevations in NE and HR simultaneously in response to the dual challenge suggest greater sympathetic activation that, in turn, could possibly explain the alteration in the distribution of lymphocyte subsets, resulting in ineffective cell-mediated immune responses [36, 37]. Therefore, these findings indicate that acute stress enhances innate immunity and possibly suppresses adaptive immunity, and these alterations can be likely enhanced at higher intensities of physical stress. The appropriate redistribution of immune cells in response to acute stressors is imperative to an effective and efficient immune response in preparation for potential invaders and injury [26, 38]. However, when exposed to chronic stress, immunoprotection can be suppressed by reducing immune cell number, function (cytotoxicity), and proliferation, thereby promoting susceptibility to diseases [39, 40]. 

Obesity is considered a chronic inflammatory condition that enhances the risk of numerous inflammatory diseases, including diabetes and cardiovascular disease (CVD). These obesity-attributable illnesses have been discovered to have a strong association with inflammatory parameters in plasma such as proinflammatory cytokines (TNF-*α* and IL-6) [41, 42]. In addition to plasma inflammatory mediators, the circulating mononuclear cells in obese individuals may be more readily stimulated to produce inflammatory cytokines [43]. Interestingly, along with physical illnesses, obesity is associated with job-associated stress and psychosocial disorders such as depression and chronic anxiety [44–46]. These stress-related disorders have been found to lead to increased risk of CVD and mortality in obese patients [47].

Chronic stress has been shown to be associated with disturbances of the HA and SA axes and is linked to abdominal adiposity [48]. In response to acute stress, elevated cortisol levels are associated with high central adiposity [49–51]. Furthermore, studies have demonstrated an increase in SA axis reactivity in obesity patients [52–54]. This occurrence seems to be pivotal to understand how stress may upregulate the inflammatory conditions in obese individuals. Recently, studies have shown that obese subjects exhibit higher proinflammatory cytokine production such as IL-6 in plasma and *ex vivo* compared with normal-weight subjects in response to acute mental stress [50, 55]. Although chronically elevated cortisol is thought to have deleterious effects on the immune system, a suppressive effect of immune regulation has been shown in response to acute stressors [56]. Importantly, Wirtz et al. [57] have revealed that individuals with higher body mass index demonstrated lower glucocorticoid sensitivity, resulting in a diminished ability to inhibit production of TNF-*α* following acute mental stress.

 In addition, *β*-adrenergic receptors have been shown to mediate catecholamine-induced decreases in proinflammatory cytokines [58, 59]. Stress has been demonstrated to downregulate beta-adrenergic receptor expression and functions on monocytes and NK cells, resulting in the elevation of TNF-*α* and IL-6 [46]. These are key proinflammatory cytokines involved in CVD, chronic anxiety, and depression [60]. Furthermore, previous studies demonstrated that increased tension-anxiety, a subscale of the Profile of Mood States (POMS), is correlated with the downregulation of *β*-adrenergic receptors [61]. Individuals with high life stress and hostility have less lymphocyte *β*-adrenergic sensitivity [62]. Taken together, these findings suggest that obesity could diminish the inhibitory effect of *β*-adrenergic receptors in response to acute stress, resulting in a greater release of proinflammatory cytokines [50, 55]. Thus far, it has been discovered that obese individuals have reduced *β*-adrenergic receptor density [63] and higher plasma NE and EPI concentrations [64]. Hence, the investigation of mechanisms of *β*-adrenergic receptor regulation to stress may provide insight into the role of psychoneuroimmunological processes in obese populations' health and disease. 

Although the underlying mechanisms contributing to the relationship of the stress response, obesity, and proinflammatory cytokines remain to be determined, elevated levels of leptin have recently been implicated as a contributing factor that links acute stress to inflammation. Leptin, an adipocyte-derived hormone, plays an important role in metabolism, adiposity, and vascular inflammation and has been implicated in the development of coronary heart disease [65]. *In vitro* stimulation of cultured human endothelial cells with leptin has induced an increased accumulation of levels of proinflammatory mediator (e.g., monocyte chemotactic protein-1) via activation of nuclear factor-kappa B [66]. Interestingly, recent research has shown that people who undergo acute mental stress demonstrate increases in leptin levels, and these increases are positively correlated with waist circumference [67, 68]. Brydon et al. [68] also showed that a positive correlation between basal circulating leptin and IL-6 exists in response to mental stress. These findings suggest that leptin may partially contribute to inflammatory response following acute stress. Future investigation should attempt to understand the mechanisms contributing to the relationship between obesity and proinflammatory reactivity to stress. In turn, an understanding of how the mind and body interact and impact health can directly influence how we develop targeted treatments, such as exercise-training and weight loss programs, as therapeutic interventions for obesity-associated cardiovascular, chronic infectious, and inflammatory neuropsychiatric diseases.

## 3. Physical Activity and Immune Function

Physical activity has long been associated with improvements in aerobic capacity [69], strength [70], muscle growth [71], and body composition [70]. However, it is now widely accepted that chronic physical activity enhances immune function and attenuates the likelihood of chronic disease, such as CVD, diabetes, and obesity [72, 73]. Initially, unaccustomed exercise places a stressor on the body resulting in fatigue [74]; however, once the recovery process occurs, beneficial adaptations are the result. In fact, fit individuals (those who partake in regular physical activity) have a lower incidence of infection compared to inactive and sedentary individuals [75, 76], suggesting that physical activity may improve the immune response. Moreover, these benefits to immune function in relation to regular exercise include decreased levels of proinflammatory cytokines TNF-*α* [77], IL-6 [78], and CRP [79] along with an increase in the anti-inflammatory marker (IL-10) [78]. Additionally, exercise is associated with decreased levels of depression [80]. To fully comprehend the positive benefits of exercise to immune function it is necessary to examine the stress and recovery response to exercise. Additional insight into how exercise affects acute and chronic inflammation is necessary to understand the importance of exercise as an antagonist to the current obesity epidemic.

### 3.1. Exercise and the Stress Response

 Intense exercise training places a stimulus on the body often resulting in myofiber damage, muscle soreness, and edema [81]. This damaging effect particularly occurs in novice trainees who are stressed by an unfamiliar stimulus. This initial fatigue in response to a new stimulus is described in Hans Seyle's landmark work, the general adaptations syndrome (GAS) [74], as the “alarm reaction stage.” Following the initial alarm response, the GAS explains that once recovery takes place, an individual enters the stage of resistance, indicating the capability of undertaking further stress. The onset of the stage of resistance signifies the adaptation to the initial stress realized in the alarm stage. This concept of initial fatigue and recovery is similar to the repeated bout effect (RBE), which states that performing the same exercise stimulus within 6 months of the initial bout results in an attenuated level of myofiber damage [81]. Consequently, the incurred adaptation is specific to the task performed in the initial exercise bout. This concept of specificity is otherwise known as the specific adaptations to imposed demands (SAID) principle, which states that individuals adapt to the specific stressor placed upon them to become more fit for living conditions [82]. Although initial fatigue leads to long-term adaptation without programmed rest and variation to exercise-training volume and intensity, tissue repair may not fully transpire and overtraining syndrome may develop [83]. Moreover, lack of ample recovery for tissue repair may result in chronic inflammation and central fatigue potentially having deleterious effects on exercise performance. Furthermore, a state of chronic inflammation, which impairs immune function, may contribute to an increased probability of obesity, CVD, and diabetes.

### 3.2. Acute and Chronic Inflammation

 When dissecting the subsequent effects of the inflammatory response, it is necessary to understand that inflammation can be both acute and chronic in nature [84]. Acute inflammation is an immediate response to stress and may not necessarily be indicative of long-term adaptations. To illustrate, acute stress hormone response, such as cortisol, has increased significantly in response to high-volume resistance training [85]. However, long-term exercise training of two years in length has resulted in decreased resting cortisol concentrations [86]. Thus, chronic exercise training appears to reduce resting cortisol levels. Therefore, acute elevations in markers of stress signify an immediate stress response; however, long-term adaptations to physical activity appear to favor parasympathetic dominance.

### 3.3. Exercise and Acute Inflammation

 A typical acute response to an infection, stressor, or immune system stimulator lipopolysaccharide (LPS) is the elevation of the proinflammatory cytokine TNF-*α* [87]. In rats that were exercised to exhaustion (an average of 102 minutes), an attenuated TNF-*α* response was measured compared to the response in nonexercised rats when administered with LPS for up to 6 hours [87]. In agreement, in human data, healthy men who performed aerobic exercise to exhaustion prior to the infusion of LPS exhibited lower levels of TNF-*α* compared to a non-exercising group [77]. Likewise, Nosaka and Clarkson [88] reported no increase in plasma levels of TNF-*α* following a bout of damaging resistance training of the elbow flexors. Interestingly, TNF-*α* levels have been reported to be significantly elevated in obese populations [89, 90]. Indeed, TNF-*α* has been established to be associated with insulin resistance, leading to obesity [91]. Ultimately, both aerobic and resistance exercise may be effective in attenuating acute inflammatory responses, which might have significant implications to preventing obesity. 

### 3.4. Exercise and Chronic Inflammation

 Numerous studies have been conducted on the relationship between exercise and concentrations of CRP [79, 92, 93]. These studies all demonstrate an inverse relationship between CRP concentrations and physical activity [92]. Further, physical fitness measured by maximal oxygen consumption (VO_2_ max) is also inversely related to CRP concentrations [94]. Data also indicates that active older men (i.e., exercising 4 days/wk) have lower levels of IL-6 and greater levels of IL-10 when compared to older men who perform a low amount of physical activity (i.e., not active most days of the week) [78]. 

 Moreover, multiple studies show that lifestyle interventions with exercise impact the inflammatory response [95–98]. Balagopal et al. [95] reported that obese adolescents who underwent a 3-month lifestyle intervention of enhanced physical activity and nutrition habits had decreased body fat percentage, insulin resistance, CRP, and IL-6. Additionally, nine months of endurance training in 14 individuals preparing for a marathon resulted in decreased levels of CRP [92]. Likewise, an exercise intervention of 3 years, which gave detailed advice in regard to physical activity, in 60 obese women resulted in weight loss along with decreased levels of TNF-*α* [99]. In fact, an 8-week exercise-training program consisting of 4 days/wk of cycling between 40 and 50% VO_2_ peak did not affect insulin sensitivity or CRP levels despite improvements in aerobic fitness and endothelial function [96]. One possible explanation for these conflicting results is that the intervention duration of 8 weeks was too short to elicit changes in insulin sensitivity and CRP levels. Ultimately, it seems that long-term exercise interventions (greater than 8 weeks) are effective in reduce the inflammation response and improve physical fitness.

### 3.5. Summary

 Obesity is a chronic inflammatory condition, which enhances the risk of CVD and is associated with various inflammatory cytokines (TNF-*α* and IL-6). When introduced as a new stressor, exercise acutely increases catabolic responses [85], resulting in muscle fatigue [74]. However, research indicates that regular participation in exercise leads to decreased systemic inflammation [93]. Indeed, Colbert et al. [72] related higher levels of physical activity to lower levels of IL-6 and CRP. Furthermore, exercise is beneficial during the aging process to decrease catabolic hormone responses [100]. Consequently, exercise seems to provide significant benefits that enhance immune function and decrease inflammation. Thus, exercise is recommended as an effective strategy to positively alter obesity-related immune function. 

## 4. Nutrition and Inflammation

### 4.1. Macronutrients: Quality and Quantity

#### 4.1.1. Energy Content

Chronic inflammation is influenced by energy balance. Acute overconsumption of energy has consistently resulted in increases in markers of inflammation [101]. These increases occur with or without weight gain, suggesting that chronic inflammation in overweight or obese individuals may be strongly influenced by caloric load and not necessarily the primary result of increased adiposity. This may also help to explain the prevalence of chronic disease in the Western world where diets often include calorie-dense foods (e.g., “fast food”). Conversely, caloric restriction and/or fasting can also result in increases in inflammation [102]. These findings suggest maintenance of energy balance as an important factor in the prevention of systemic inflammation. Interestingly, outside of energy balance, the type of macronutrients that we consume also plays a pivotal role in whole-body inflammation.

#### 4.1.2. Carbohydrates

Carbohydrates (CHO) can dramatically influence whole-body inflammation. In addition, there are many factors that affect the inflammatory potential of CHO intake including glycemic index (GI), glycemic load (GL), and dietary fiber. The degree to which a CHO increases the blood sugar response (high-GI equals greater increases in blood sugar) will influence the inflammatory response. In fact, GI has consistently shown a positive correlation with biomarkers of whole-body inflammation. Specifically, markers of inflammation increase acutely following a high GI meal [103]. Of note, markers of inflammation either decline or remain unchanged following low-GI meals [104]. This discrepancy may be a function of GL, the product of dietary GI, and quantity of CHO actually eaten, as consumption of a large quantity of low-GI food has been shown to increase markers of inflammation to a similar degree as to the consumption of a small quantity of high-GI food [105]. However, data on the relationship between GL and inflammation are conflicting. Although some studies have shown a positive correlation between dietary GL and markers of inflammation (i.e., C-reactive protein, CRP) [106, 107], others have failed to report a significant association [108]. This divergence may be due to differences in energy content of the diets in GL studies, which, as discussed above, has been shown to have a strong relationship with inflammation [109]. Many GL studies compare varying dietary CHO amounts while simultaneously limiting calories to encourage weight loss. Therefore, these alterations may go against each other and possibly explain the confounding results. 

Increased consumption of refined carbohydrates in the modern diet has not only led to increased consumption of high-GI diets but also reduced intake of dietary fiber. This low fiber intake may explain the increased prevalence of chronic inflammation and disease as numerous studies have reported an inverse relationship between fiber intake and CRP [110]. Of interest, the anti-inflammatory effects of fiber appear to hold true for both dietary fiber and fiber supplements [111]. Moreover, this relationship may also hold true for insoluble fibers, which have been shown to positively affect immune function [112]. Dietary fiber may also influence inflammation through its mediating effects on glycemia. In one lifestyle intervention study on a representative sample of Italian adults, blood CRP concentrations were lowered along with fasting blood glucose in response to increased fiber intake, independent of weight loss [113]. Thus, perhaps by mediating the absorption of nutrients and modulating changes in blood sugar, fiber effectively reduces inflammation.

#### 4.1.3. Fats

The inflammatory potential of fat is a function of the type of fat being consumed. Numerous studies have implicated saturated fats (SFAs) as inflammatory agents. SFAs, which are commonly found in processed meat, refined grain, and/or fried foods, have been shown to increase markers of inflammation such as CRP, IL-6, and E-selectin, a vascular adhesion molecule [114]. A strong correlation between SFAs and CRP can be found in the National Health and Nutrition Education Survey (NHANES 99-00) [115]. Furthermore, Arya et al. [116] reported that among other dietary nutrients, SFA levels were the most important predictor of CRP levels. However, it should be noted that some studies have failed to show a correlation [117] or, in some cases, have even shown a slight negative correlation between SFA and CRP levels [118]. This discrepancy may be due to the population studied and confounding variables such as other nutrients included in the diet, physical activity, and the population studied. Trans fats (TFA), fatty acids containing one double bond created via hydrogenation of vegetable oils, which are found in foods such as butter, margarine, milk fat, and fried foods, may also increase markers of inflammation. In the Nurses' Health Study, a strong correlation was shown between TFA levels and inflammatory biomarkers of CRP, IL-6, and E-selectin [119]. This may explain the strong connection between serum TFA levels and coronary heart disease [120].

Conversely, monounsaturated fatty acids (MUFAs) and polyunsaturated fatty acids (PUFAs) have an inverse relationship with the inflammatory biomarkers. Levels of certain PUFAs such as omega-3 fatty acids, common in cold-water fish, are consistently found to have an inverse relationship with IL-6 [121]. The effects of other common PUFAs such as omega-6 fatty acids are less clear. Inflammatory and noninflammatory effects have been reported with omega-6 consumption [122]. Indeed, the effectiveness of PUFAs in reducing markers of inflammation may lie in the ratio between omega-3 and omega-6 PUFAs. It has also been suggested that a higher ratio of omega-3 to omega-6 PFAs increases the anti-inflammatory potential of these fats [123]. MUFAs also appear to be anti-inflammatory as several studies [124, 125], but not all [118], have reported an inverse relationship between MUFA levels and inflammatory markers. Stronger evidence for this inverse relationship is found specifically between olive oil consumption, which contains high levels of MUFAs, and systemic inflammatory markers [126].

#### 4.1.4. Protein

The effects of proteins on inflammation seem to vary depending on the source of the protein. Red meat is typically considered as proinflammatory. This is likely due to the association between high dietary red meat intake and both coronary heart disease [127] and type II diabetes [128]. However, evidence from studies on lean red meat has failed to show any increase in markers of inflammation [129]. This suggests that the quality of the meat may be more indicative of its inflammatory potential. Indeed, consuming processed meat (e.g., bacon, hamburger, and sausage) seems to increase the risk of type II diabetes, which is associated with increased whole-body inflammation [130]. 

Inflammation from protein ingestion may also vary between meat-based, plant-based, and milk-based proteins. In a study from Denmark, obese participants were fed a single high-fat meal containing 4 different sources of protein: fish (cod), whey isolate, gluten, or casein. Serum levels of inflammatory cytokines were monitored in the 4 hours following the meal. Cod protein and gluten resulted in a blunted increase in markers of inflammation versus the whey meal but were not different from the casein meal [131]. Therefore, consuming cod protein and/or plant-based protein may be beneficial for whole-body inflammation relative to some milk-based proteins (whey). Evidence also exists to support the use of soy protein to blunt inflammation. In fact, reductions in markers of inflammation have been shown with consumption of soy [132], soybean oil [133], and soy nuts [134]. More data is needed, however, before a definitive conclusion can be made for which types of proteins may work best to reduce whole-body inflammation. 

### 4.2. Micronutrients/Antioxidants

#### 4.2.1. Flavonoids

Flavonoids, naturally occurring antioxidant, commonly found in foods such as onions, apples, grapes, berries, and cocoa, are anti-inflammatory agents. Common flavonoids such as quercetin, kaempferol, malvidin, peonidin, daidzein, and genistein have been reported to have an inverse relationship with CRP [135].

#### 4.2.2. Carotenoids

Carotenoids are the natural pigments that give many fruits and vegetables their distinctive bright colors. These are found in abundance in oranges, sweet potatoes, kale, and spinach. Carotenoids such as alpha-carotene, beta-carotene, and lutein seem to have anti-inflammatory potential. In fact, participants of one study with the highest serum levels of alpha-carotene were significantly less likely to have high levels of IL-6. Interestingly, the opposite was also found to be true where low levels of various carotenoids in the blood were associated with increased levels of IL-6 [136]. Furthermore, another study reported a potentially protective effect of lutein and lycopene against atherosclerosis, which the authors report to be likely a result of a reduction in inflammatory markers [137].

#### 4.2.3. Magnesium

Magnesium, found typically in whole grain, green leafy vegetables, nuts, and legumes, may be one of the most effective agents for combatting chronic inflammation. Numerous studies have reported the inverse relationship between magnesium intake and CRP and IL-6 [119, 138], in some cases in a dose-dependent manner [138]. 

#### 4.2.4. Vitamins

Vitamins, found commonly in fruits and vegetables, are essential to immune function and are thus a necessary impediment to excessive inflammation. Numerous vitamins such as vitamins C, E, A, B6, and riboflavin each have been associated with reduced cytokine production (e.g., IL-6) [139]. 

### 4.3. Whole Unprocessed Foods

#### 4.3.1. Fish

In general, consumption of fish has been linked to various health benefits, particularly heart health [140]. Some of these health benefits may be due to reductions in markers of inflammation [141]. This seems likely as certain fish, particularly cold-water fish like salmon and mackerel, contain high levels of PUFAs like omega-3 fatty acids, which as discussed above, likely have anti-inflammatory effects. Indeed, the anti-inflammatory nature of fish seems to be a direct result of PUFA content as numerous studies on fish oil supplements (high in omega-3 PUFAs) have reported reductions in markers of whole-body inflammation [142, 143]. In agreement, using salmon fed 3 different diets to manipulate the omega-3 fatty acid content of the fish, it was found that only the participants consuming salmon containing the highest levels of omega-3s saw reductions in IL-6 [144]. 

#### 4.3.2. Fruits and Vegetables

A large body of research exists documenting the many health benefits of consuming fruits and vegetables [140]. Moreover, the anti-inflammatory effects of consuming fruit and vegetables are well documented [145, 146]. This is no surprise considering that fruits and vegetables contain high concentrations of dietary fiber, flavonoids, carotenoids, and vitamins, which have all been associated with reductions in markers of inflammation, as discussed above. Beyond these ingredients, certain fruits contain particular compounds such as anthocyanins and bromelain, which may also be anti-inflammatory [147–151]. Anthocyanins are the pigments found in fruits such as strawberries, cherries, blackberries, and black currants. Research has indicated the potential of anthcyanins to inhibit cancer formation in rodents [147]. This finding could be the result of reported anti-inflammatory properties of anthocyanins. A recent study on participants with high blood cholesterol levels reported that consumption of an anthocyanin supplement twice daily for 24 weeks resulted in reductions in CRP and other markers of systemic inflammation [148]. 

Bromelain is a proteolytic enzyme found primarily in pineapples. Among many other therapeutic benefits (e.g., improved endothelial function, enhanced absorption of antibiotic drugs, and inhibition of tumor cell growth), bromelain is an effective anti-inflammatory agent [149]. These anti-inflammatory properties have been observed *in vitro* via its modulating effect on certain inflammatory cytokines [150]. The benefits are also apparent in clinical trials which report the effectiveness of bromelain for treating certain inflammation-derived diseases/conditions (e.g., osteoarthritis) [151].

### 4.4. Beverages

#### 4.4.1. Tea/Coffee

Tea consumption is associated with reduced markers of inflammation. In fact, after 6 weeks of black tea consumption in healthy men, there was a reduction in CRP and platelet aggregation [152]. Green tea may have similar properties as it has been indicated to be an effective antioxidant *in vitro *[153]. However, the effectiveness of green tea at reducing markers of inflammation like CRP in clinical trials is less clear. Studies examining 1-month to green tea consumption on healthy males [154] and male smokers [155] have failed to report an effect on CRP levels.

The effects of coffee consumption on inflammation are equivocal. Some studies have reported anti-inflammatory effects [156], while others have reported no effects at all [157]. This could be due to genetic factors as a recent study revealed a genetic polymorphism affecting one's responsiveness to caffeine [158]. Because caffeine may have anti-inflammatory properties [159], the effectiveness of coffee as an anti-inflammatory agent may be mediated by one's responsiveness to caffeine. While speculative, the impact of physical activity and other lifestyle factors likely plays a critical role in the overall anti-inflammatory response to these nutrients.

#### 4.4.2. Alcohol

The effectiveness of alcohol at reducing or preventing inflammation seems to depend on the amount consumed. While excessive consumption and/or binge drinking is known to have many adverse health outcomes including increased inflammation [160], moderate intake (e.g., ~150 mL of wine per day) of alcohol seems to reduce markers of inflammation in various populations [161]. The degree to which these markers are reduced may depend on the source of alcohol. Red wine, for example, has consistently been reported to have anti-inflammatory effects [162], leading to speculation that these effects may simply be derived from grape-derived antioxidants in the wine. However, other alcoholic beverages such as beer and liquor have also been found to have anti-inflammatory properties [163]. Overall, this implicates small doses of alcohol to be anti-inflammatory. In support, no effects on markers of inflammation were reported with consumption of nonalcoholic beer [164]. 

### 4.5. Herbs and Spices

Many herbs and spices have been reported to have anti-inflammatory effects [165]. For example, cloves, garlic, curcumin, cinnamon, fennel, ginger, fenugreek, ginseng, and capsaicin have all shown to be effective at reducing or preventing inflammation. However, the full explanation of these herbs and spices with regard to inflammation has already been conducted [165]. 

### 4.6. Summary

It is apparent that nutrition plays a critical role in the whole-body inflammatory response. Indeed, overconsumption of highly processed foods and lack of fruit and vegetable intake are common in North America [166] and parallel the increase in obesity and other inflammatory-associated diseases. The literature suggests that an energy-balanced nutrient intake combined with low-GI and low-GL CHO, higher intake of omega-3 PUFAs, a focus on lean meat consumption, and a greater intake of fruits and vegetables may provide the ideal anti-inflammatory environment. A nutritional program that offers these macronutrient choices would also likely supply beneficial vitamins, minerals, and other micronutrients to aid in the anti-inflammatory process.

## 5. Conclusion

 It is evident that lifestyle factors, such as stress, physical activity, and nutrition, both cooperatively and independently influence the inflammatory profile associated with excessive adiposity and may play a critical role in the development or prevention of obesity-related comorbidity. Chronic stress, sedentary behaviors, and overnutrition are lifestyle factors conducive to obesity and systemic inflammation. On the other hand, stress reduction and proper nutritional and exercise programming have each shown to be beneficial in ameliorating the inflammatory processes associated with obesity whether they are directly or indirectly involved with weight loss management. 
